# Supplementary material for: Light enhanced calcification in Stylophora pistillata: effects of glucose, glycerol and oxygen
Source: PeerJ. 2014 May 13;2:e375. doi: 10.7717/peerj.375 (PMC4034610; doi:10.7717/peerj.375)
Supplement: Supplemental Information — Supplemental material containing additional methods for estimating flow speeds, a comparison of ATP extraction protocols, summary tables for ATP extractions and for control values, and an additional figure to illustrate the incubation chambers. [file peerj-02-375-s001.doc]

Supplemental materials

Stirring speed

To estimate how mass transfer rates achieved in our incubation system relate to flow speed, gypsum dissolution experiments were carried out (e.g. Dennison and Barnes 1988; Falter et al., 2005). Plaster cylinders ~13 mm diameter x 28 mm long were suspended on nylon line, as done for coral fragments. Dissolution rates were measured in 250 ml flasks stirred with 7.8 x 39.2 mm stir bars at 145 rpm and in small scale flumes at different flow speeds. A flow speed of ~8 cm s^-1^ yielded dissolution rates similar to those obtained in the flasks.

ATP extraction optimization

As multiple extraction protocols exist for ATP extraction, but no comparison of methods has been published for corals, several methods were initially tested. The sulfuric acid extraction protocol (described below) was ultimately adopted for use with experimental corals.

Extractions were carried out on two different days – all extraction protocols (except Tris-EDTA) were used each day extractions were carried out with at least five microcolonies for each extraction protocol each day. Microcolonies were assigned to a given extraction protocol haphazardly. Extractions were carried out in 50 ml conical polypropylene tubes (Falcon) to which 14-15 ml (actual amount weighed to the nearest milligram) of the given extraction solution (see below) had been added. This volume of extraction solution was sufficient to completely submerge the microcolony when placed in the tube. In addition to microcolonies, blanks, seawater, and ATP standards were subjected to the same extraction protocols to verify zero values, potential effect of seawater contamination, and percent recovery.

Sulfuric acid

ATP extraction with sulfuric acid was carried out as described in the text.

Trichloroacetic acid

Extraction with trichloroactic acid (TCA) followed a protocol adapted from Larsson and Olsson (1979). Microcolonies were placed in 2.5% w/v TCA with 2 mM EDTA (kept on water/ice prior to use) and placed immediately in a sonicator (Branson 200 Ultrasonic Cleaner) filled with water/ice. Samples were sonicated for 15 min, tubes with microcolonies re-weighed, and a sample of the extract transferred to a 1.5 ml polypropylene tube (Eppendorf) and stored at -80 ^o^C until measured. The remaining extract was used for protein extraction (see below).

NaOH

ATP extraction with NaOH followed a protocol adapted from Al-Horani et al. (2003). Microcolonies were placed in 1 M NaOH with 50 mM EDTA (pre-heated to 90 ^o^C) and incubated at 90 ^o^C for 15 min. A sample of the extract was transferred to a 1.5 ml polypropylene tube and stored at -80 ^o^C until measured for ATP. A second sample of the extract was taken for protein measurement and stored at -20 ^o^C.

Boiling buffers

ATP extraction with 20 mM Tris-acetate (7.75) or 20 mM Tris-acetate with 2 mM EDTA pH 7.75 followed a protocol adapted from Larsson and Olsson (1979). Microcolonies were placed in the given buffer (pre-heated in a boiling water bath) and incubated in the boiling water bath for 2-5 min (ATP contents were similar for 2 and 5 min incubations so data were pooled). A sample of the extract was transferred to a 1.5 ml polypropylene tube and stored at -80 ^o^C until measured. The remaining extract was used for protein extraction (see below).

ATP measurement

Prior to measurement, samples were thawed on water/ice, samples extracted in acid were neutralized with Tris (base), samples extracted in NaOH were neutralized with acetic acid and all samples were diluted 1:1250 with 20 mM Tris-acetate pH 7.78.

ATP measurements were made using a luciferin/luciferase based ATP detection kit (Roche ATP Bioluminescence Assay Kit HS II) with light emission measured using either a Lumat 9507 or 9508 luminometer (Berthold). ATP standards were diluted in 20 mM Tris-acetate pH 7.78 to prepare a standard curve and to prepare a ~50 ng ATP ml^-1^ solution which was used for standard additions (0, 10, 20, or 30 μl added to 450 μl of diluted extract) and for verifying samples did not inhibit the luciferase reaction. The measurement sequence was as follows: a 75 μl sample was added to a polystyrene tube and measured for a 2 s background count, 75 μl injection of the luciferase reagent, 3 s delay, 4 s count, 20 μl (25 μl for the Lumat 9507) injection of an ATP standard, 4 s delay, 4 s count. The second injection of ATP was used to verify samples were not inhibiting the reaction. All measurements were made in duplicate. ATP concentrations were calculated based on standard addition.

Normalizations:

Protein and surface area were measured as described in the methods.

For a subset of microcolonies used for comparing ATP extraction protocols (5-10 per protocol) post protein extraction, skeletons were soaked overnight in seawater and reweighed (buoyant weight) to estimate mass loss (all having been weighed via buoyant weight prior to ATP extraction). All coral skeletons were rinsed with fresh water and air dried prior to measuring dry weight, however for data presented in Table S1, calculated dry weights (based on buoyant weights prior to extraction) are used so skeletal loss during extraction does not bias the interpretation.

All ATP extraction comparison data were analyzed using a one-way ANOVA model which included the day on which extractions were carried out as a blocking variable, a Scheffe multiple comparison procedure was used for pairwise comparisons.

ATP extraction comparison

The microcolonies used for ATP extractions had similar dry weights, surface areas, protein contents and growth rates, no significant (p>0.2) differences were found among the microcolonies used for each extraction protocol in these parameters (Table S1). ATP contributed by endolithic organisms was not a significant concern as extractions of corals from which the tissue layer had been removed via waterpik had ATP levels that were ~1% of those in intact specimens. Based on dry weights calculated from buoyant weight, corals extracted with Tris buffers lost, on average, less than 0.5% of their weight, corals extracted with TCA and NaOH lost ~4% of their weight, and corals extracted with H_2_SO_4_ lost ~7.5% of their weight. These estimates likely over-estimate the mass lost during extraction as no attempt was made to remove bubbles from within the skeleton following extraction.

For raw ATP content as well as all normalizations of ATP data, differences among ATP extraction methods were significant (p<0.001). Extraction with sulfuric acid yielded significantly (p<0.05) more ATP than any other extraction protocol regardless of the method used for normalizing the data with the exception of trichloroacetic acid when normalized via skeletal weight for which the difference was not statistically significant. Extraction with TCA or NaOH yielded significantly higher (p<0.05) ATP yields than extraction with Tris regardless of normalization method. No significant differences were found between extractions with TCA or NaOH.

The results of our initial tests of extraction protocols (Table S1) suggest that a sulfuric acid extraction protocol (based on Fang et al. 1987, 1991) has consistently higher yields of ATP than the other tested protocols and thus may be appropriate for future measurements. However, where it is desirable to measure skeletal parameters (surface area, dry weight, etc) on the same samples, the sulfuric acid extraction solution must be neutralized rapidly on completion of the extraction to avoid excessive dissolution of the skeleton. The NaOH extraction protocol employed by Al-Horani et al. (2003) provides an alternative in which skeletal loss is less of an issue, though ATP yields were lower. In addition to the methods for which detailed testing was carried out, a phenol extraction protocol based on Chida et al (2012) was also tested (using sonication in phenol instead of sulfuric acid (described above) to extract ATP), however yields on similarly sized specimens were ~50% of those obtained with sulfuric acid, so further testing was abandoned.

Table S1. Number of replicates (N), average values and standard deviations are given for the corals used with each ATP extraction method for the various parameters measured (dry weight, surface area (SA), protein, growth rate, and ATP content) as well as for the ATP content normalized to the other measured parameters.

Table S2. Average values with standard deviation in parenthesis for protein, skeletal dry weight, surface area (SA), protein per unit surface area, and calcification and respiration rates of control corals normalized to each of the normalization parameters for bleached and unbleached microcolonies. N=32 for bleached, N=43 for zooxanthellate for protein, dry weight, and SA; N=15 for bleached and N=19 for zooxanthellate for respiration and calcification.

| Parameter | Bleached | Zooxanthellate |
| --- | --- | --- |
| Protein (mg) | 12.3 (4.0) | 17.2 (4.8) |
| Dry weight (g) | 1.6 (0.9) | 1.6 (0.5) |
| SA (cm^2^) | 6.2 (1.7) | 6.6 (1.4) |
| Protein/SA (mg/cm^2^) | 2.0 (0.5) | 2.6 (0.5) |
| μmol O_2_/d/mg protein | 0.83 (0.29) | 5.2 (1.2) |
| μmol O_2_/d/g dry weight | 6.2 (1.4) | 51 (10) |
| μmol O_2_/d/cm^2^ | 1.6 (0.3) | 12.5 (1.8) |
| μmol CaCO_3_/d/mg protein | 0.46 (0.2) | 10.2 (3.5) |
| μmol CaCO_3_/d/g dry weight | 3.6 (1.5) | 96 (21) |
| μmol CaCO_3_/d/cm^2^ | 0.92 (0.38) | 23.7 (4.2) |

Figure S1. Diagram representing the incubation chamber used for the experiments, drawn as a cross section through the center.
